# Herbivory-responsive calmodulin-like protein CML9 does not guide jasmonate-mediated defenses in *Arabidopsis thaliana*

**DOI:** 10.1371/journal.pone.0197633

**Published:** 2018-05-16

**Authors:** Monika Heyer, Sandra S. Scholz, Dagmar Voigt, Michael Reichelt, Didier Aldon, Ralf Oelmüller, Wilhelm Boland, Axel Mithöfer

**Affiliations:** 1 Department of Bioorganic Chemistry, Max Planck Institute for Chemical Ecology, Jena, Germany; 2 Department for Plant Physiology, Matthias Schleiden Institute, Friedrich Schiller University, Jena, Germany; 3 Institute for Botany, Technical University Dresden, Dresden, Germany; 4 Department of Biochemistry, Max Planck Institute for Chemical Ecology, Jena, Germany; 5 UMR 5546 CNRS-Université Toulouse III, Pôle de Biotechnologie Végétale, Castanet-Tolosan, France; Estacion Experimental del Zaidin, SPAIN

## Abstract

Calcium is an important second messenger in plants that is released into the cytosol early after recognition of various environmental stimuli. Decoding of such calcium signals by calcium sensors is the key for the plant to react appropriately to each stimulus. Several members of Calmodulin-like proteins (CMLs) act as calcium sensors and some are known to mediate both abiotic and biotic stress responses. Here, we study the role of the *Arabidopsis thaliana* CML9 in different stress responses. CML9 was reported earlier as defense regulator against *Pseudomonas syringae*. In contrast to salicylic acid-mediated defense against biotrophic pathogens such as *P*. *syringae*, defenses against herbivores and necrotrophic fungi are mediated by jasmonates. We demonstrate that *CML9* is induced upon wounding and feeding of the insect herbivore *Spodoptera littoralis*. However, neither different *CML9* loss-of-function mutant lines nor overexpression lines were impaired upon insect feeding. No difference in herbivore-induced phytohormone elevation was detected in *cml9* lines. The defense against the spider mite *Tetranychus urticae* was also unaffected. In addition, *cml9* mutant lines showed a wild type-like reaction to the necrotrophic fungus *Alternaria brassicicola*. Thus, our data suggest that CML9 might be a regulator involved only in the defense against biotrophic pathogens, independent of jasmonates. In addition, our data challenge the involvement of CML9 in plant drought stress response. Taken together, we suggest that CML9 is a specialized rather than a general regulator of stress responses in *Arabidopsis*.

## Introduction

The environment of organisms is continuously changing over their lifetime. Whereas most of the organisms are able to escape unfavorable conditions, plants are sessile and need to cope up with these changes. Thus, they developed a plenty of strategies to overcome biotic as well as abiotic stresses throughout evolution [1–3]. Since abiotic and biotic alterations of the environment often occur simultaneously, a complex signaling network is coordinating all the different plant stress responses. Phytohormones play an essential role in these signaling pathways, such as abscisic acid (ABA) as key regulator of abiotic stress responses and salicylic acid (SA) and jasmonic acid (JA) as main mediators of biotic stress responses [3–5]. Resulting from the multiplicity of environmental changes, these stress-related phytohormone pathways overlap. For instance, defense reactions against the specialist herbivore *Pieris rapae* L. (Lepidoptera, Pieridae) and the generalist *Spodoptera littoralis* Boisd. (Lepidoptera, Noctuidae) are co-regulated by ABA and JA [6]. Plant-pathogen interactions are highly influenced by abiotic conditions and thus regulated by ABA as well [7]. Besides, the crosstalk between JA and SA has been largely investigated and several examples in herbivory and pathogen defense are known [3, 6, 8, 9].

Upstream of the phytohormone network, changes in the intracellular calcium (Ca^2+^) level are one of the earliest signaling events after treatment with various environmental stimuli [10]. Depending on the stimulus different calcium signatures can be measured that vary in their location in the cell as well as in their dynamics [11]. To react appropriately to each stimulus, decoding of the particular calcium signature is necessary. The first step in translating the calcium code into a stress response is the recognition of the Ca^2+^ by sensor proteins. Calcium sensors are proteins that are able to bind Ca^2+^ via a helix-loop-helix structure, called EF-hand [12]. Two classes of calcium binding proteins are distinguished: sensor relays and sensor responders. Calcium sensor responders have an enzymatic function additional to their EF-hands that is activated upon binding calcium and by this initiating further signal transduction. In contrast, sensor relays have no other functional domain besides the EF-hands. By binding Ca^2+^ their conformation is changed, so that an interaction with the respective targets is possible [11]. In the model plant *Arabidopsis thaliana* (L.) Heynh., 250 calcium sensing proteins are identified, including sensor relays like calmodulins (CAMs), calmodulin-like proteins (CMLs) and calcineurin B-like proteins (CBLs) and sensor responders like Ca^2+^-dependent protein kinases (CPKs) [13].

Among them, the group of CMLs is of particular importance for the plant calcium decoding, since they are unique for plants. Several studies revealed that they play a role in calcium perception in a wide range of plant stress responses. *CML24* is known to be regulated upon touch, extreme temperatures, darkness, ABA and H_2_O_2_ treatment and it is a regulator of salt stress response [14, 15]. *CML11*, *CML12*, *CML16*, *CML17* and *CML23* are induced by elicitors in insect oral secretions (OS), suggesting their possible role in defense against herbivores [16]. Furthermore, some CMLs are known to regulate abiotic as well as biotic stress responses and by this being of special interest in understanding the complex signaling network of the plant. Recently it was shown that CML37 and CML42 are regulators of plant defense against the herbivore *S*. *littoralis* and of drought stress. Whereas CML37 is a positive regulator of both stress responses, CML42 acts antagonistically to CML37 [17–19]. Besides, CML37 is induced upon infection with the (hemi)biotrophic pathogen *Pseudomonas syringae* (van Hall) as well [20]. Another CML coordinating different stress responses is CML9, also known as CAM9. It was suggested to be a negative regulator of the ABA pathway during seed germination and seedling growth. In addition, knock-out of *CML9* was leading to a higher salt and drought stress tolerance of the adult plants [21]. On the other hand, *CML9* was shown to be upregulated upon *P*. *syringae* infection and treatment with SA [22, 23]. Depending on the bacterial strain, CML9 was acting either as a positive or negative regulator of the plant immune reactions [23]. Additionally *CML9* is induced by mechanical stimuli and by *S*. *littoralis* OS [14, 16, 21], supposing that it might also play a role in herbivore defense.

In order to verify this hypothesis, we studied the functional relevance of CML9 in the defense against the insect herbivore *S*. *littoralis* and the spider mite *Tetranychus urticae* Koch (Trombidiformes, Tetranychidae). We show here that *CML9* is rapidly induced upon feeding of the insect. The employment and analysis of *CML9* knock-out and overexpression mutants revealed that CML9 does not regulate the defense against these herbivores. To gain further insight into the putative role of CML9 in abiotic and biotic stress responses, we also investigated, if CML9 might regulate the defense against the necrotrophic pathogen *Alternaria brassicicola* (Schwein.) Wiltshire, and reexamined its function in drought stress reactions. Our data indicate that none of which is regulated by CML9. Thus, a role for CML9 as a coordinator of abiotic and biotic stress responses has to be reconsidered.

## Material and methods

### Plant growth

*A*. *thaliana* ecotype Col-0 and knock-out mutant lines *cml9-a* (SALK_006380C) and *cml9-b* (SALK_126787C; intronic T-DNA insertion lines, Col-0 background), obtained from Nottingham Arabidopsis Stock Center (NASC, Nottingham, United Kingdom), were used for the experiments. Additionally, feeding assays were repeated using the knock-out lines *cml9-1* (intronic T-DNA insertion, Col-8 background) and *cml9-2* (exonic T-DNA insertion, Ws-4 background) [21] and the overexpression lines OE-CC-2 and OE-CC-5 (Col-8 background) [23]. *A*. *thaliana* ecotypes Col-8 and Ws-4 were used as wild type, respectively. Most of the experiments were performed at MPI CE Jena. Seeds were sown in round pots with 10 cm diameter and kept at 4°C for 2 d. After stratification, plants were grown under short day conditions (10 h : 14 h, light : dark) in a growth chamber at 21°C and 50–60% humidity. FLUORA^®^ bulbs (OSRAM, Garching, Germany) were used as light source and kept in 30 cm distance to the plants to achieve a light intensity of 100 μmol m^-2^ s^-1^.

*T*. *urticae* assays were performed at the TU Dresden. Plants were grown under the described conditions at MPI CE Jena in 7 cm x 7 cm rectangular pots to allow monitoring of spider mite development. At the age of 4 weeks, plants were transferred to Dresden into a growth cabinet with slightly changed conditions. Humidity was 60–70%, temperature was around 19°C, and a mixture of F32T8/TL741/Alto and F17T8/TL741/Alto bulbs (Philips, Hamburg, Germany) was used as light source with same light intensity as FLUORA bulbs.

*Alternaria* treatments were performed at the FSU Jena. Plants were cultivated as described in Johnson, Sherameti [24]. After 10 days, plants were transferred to soil and further incubated under the published short day conditions with 9 h photoperiod.

### Insect and spider mite rearing

*S*. *littoralis* larvae were hatched from eggs (Bayer Cropsience, Monheim, Germany) and reared on an artificial diet consisting of 500 g ground beans, 1.2 L water, 9 g vitamin C, 9 g 4-ethylbenzoic acid, 9 g vitamin E Mazola oil mixture (7.1%), 4 mL formaldehyde, 1 g β-sitosterol, 1 g leucine, 10 g AIN-76 vitamin mixture, 200 mL agar solution (7.5%) (modified after Bergomaz and Boppré [25]). Insects were grown at 23–25°C with a photoperiod of 14 h.

*T*. *urticae* females (Weixdorf population) were provided by D. Voigt, TU Dresden, Germany. They were kept on *Phaseolus vulgaris* (L.) ′Valja′ and ′Saxa′ plants (ISP-International Seeds Processing GmbH, Quedlinburg, Germany) at 22–24°C, 50–65% humidity and a 16 h : 8 h light : dark cycle.

### Growth and maintenance of fungi

*A*. *brassicicola* (FSU-218) was obtained from Jena Microbial Resource Centre, Jena, Germany. The fungus was grown on potato dextrose agar (PDA) medium (pH 5.6) at 22 ± 1°C in a temperature-controlled chamber in the dark and 75% relative humidity for 2 weeks.

### Plant treatments

5- to 6-week old plants were used for all herbivory-associated experiments. Insect biomass assays, short term feeding assays with *S*. *littoralis* larvae and OS treatment were performed according to Scholz, Vadassery [18]. MecWorm [26] was used for continuous mechanical wounding of the plant. Six different shaped areas (rectangular and circular), each lasting 30 min using a speed of 12 punches per minute, were designed to realize the given time points. Areas were arranged over the leaf lamina without wounding the midrib. Tissue samples for later extraction were immediately frozen in liquid nitrogen. For investigation of the possible interaction of ABA and wounding, plants were sprayed with a 100 μM ABA solution, containing 0.02% of ethanol. After 1 h, plants were treated with MecWorm for 30 min. Time points and ABA concentrations were chosen according to Magnan, Ranty [21] and prior results in this study.

For spider mite performance assay each plant was infested with one 3- to 5-day-old adult female. Mites were kept for 2 days on the plants to allow oviposition and were removed afterwards. Plants were monitored daily over development of one generation of spider mites. Number of eggs and immatures were counted and fitness parameters calculated.

For fungi treatment *A*. *brassicicola* spore suspension was prepared as follows: 5 ml of sterile-filtrated 0.01% Tween-20 were dropped on plates of 2-week old *A*. *brassicicola* cultures and plates were gently pivoted. Remaining spores were carefully removed with a spatula. The spore suspension was washed 3 times with 0.01% Tween-20 and filtrated through a 75 μm nylon membrane. The spore concentration was determined using a haemocytometer and was adjusted to 1 x 10^6^ colony forming units (cfu) ml^−1^. Mature leaves of 5- to 6-week-old *Arabidopsis* plants were used for inoculation assay. Single leaves were detached and placed in petri dishes containing sterilized filter paper soaked with 1.5 ml water. 2 μl of *A*. *brassicicola* spore suspension or solvent control was inoculated to each leaf. Plates were sealed to keep high humidity and incubated under continuous light as described above. To analyze the viability of treated leaves, chlorophyll fluorescence parameters were measured using a FluorCam FC 800-C (PSI, Brno, Czech Republic). Before measurement, the sealed plates were incubated in the dark for 20 min. Afterwards, plates were placed into the FluorCam and analyzed using following settings: Act 1: 50%, Act 2: 50%, Super: 100%. The QY_max (maximum PSII quantum yield) was recorded.

The drought stress assay was done with four week old plants as described in Scholz, Reichelt [19]. First drought period was extended to 11 days according to Magnan, Ranty [21]. After 11 days plants were watered till soil was fully soaked, followed by a second drought period of 1 week. To avoid competition between wild type and mutant plants, each was kept in single pots. Pots were placed randomly on the same tray, to minimize experimental variation.

### Quantitative real time (qRT)-PCR

RNA from single leaves was isolated using TRIzol^®^ Reagent (Invitrogen^TM^, Darmstadt, Germany) according to the manufacturers’ instructions with slight modifications. Leaf material was ground using 2010 Geno/Grinder^®^ (SPEX^®^SamplePrep, Metuchen, USA) with a precooled cryoblock. All centrifugation steps were performed at 4°C and 16000 x g. After adding TRIzol^®^, samples were incubated for 20 min at room temperature. 300 μL chloroform was added, followed by incubation on ice for 20 min. The samples were centrifuged for 30 min. The aqueous phase was transferred into 600 μL isopropanol and samples precipitate overnight on -20°C. To pellet the RNA, samples were centrifuged for 30 min. Pellet was washed with 80% ethanol and air dried. The dried pellet was dissolved in 80 μl preheated water. RNA was treated with TURBO DNase (TURBO DNA-*free*^TM^ Kit, Invitrogen^TM^, Darmstadt, Germany) to avoid DNA contamination. RNA concentration was measured with a photospectrometer and 1 μg of RNA was transcribed into cDNA using Omniscript^®^ Reverse Transcription Kit (Qiagen, Hilden, Germany) and Oligo(dT)_12-18_ Primer (Invitrogen^TM^, Darmstadt, Germany). *RPS18B* was used as housekeeping gene and primers published for *RPS18B* and *CML9* [16] were used for expression analysis of *CML9* after herbivore-associated stimuli. For quantification of exon1-exon3 fragment of *CML9*, primers (see S1 Table) producing a product of 168 bp, were designed in NCBI Primer-BLAST (http://www.ncbi.nlm.nih.gov/tools/primer-blast/) and cross checked in Vector NTI^®^ *Express* 1.2.0 software (Thermo Fisher Scientific^TM^, Schwerte, Germany). QRT-PCR was performed in 96 well plates in a CFX96 Touch^TM^ Real-Time PCR System (Bio-Rad, München, Germany). Brilliant II QPCR SYBR green Mix (Agilent, Waldbronn, Germany) was used as master mix. The normalized fold expression was calculated with the ΔΔCP method [27]. Untreated plants or, in case of ABA spray, plants sprayed with 0.02% ethanol were used as controls and their expression level was defined as 1.

### Phytohormone quantification

Phytohormone analysis was performed according to Jimenez-Aleman, Scholz [28] with modifications. Approximately 250 mg ground plant tissue was extracted with 1.5 mL methanol containing 60 ng D_6_-abscisic acid (Santa Cruz Biotechnology, Santa Cruz, U.S.A.), 60 ng of D_6_-jasmonic acid (HPC Standards GmbH, Cunnersdorf, Germany), 60 ng D_4_-salicylic acid (Sigma-Aldrich) and 12 ng of jasmonic acid-^13^C_6_-isoleucine conjugate as internal standard. LC- MS/MS measurements were performed on an Agilent 1200 HPLC system (Agilent, Waldbronn, Germany) coupled to an API 5000 tandem mass spectrometer (Applied Biosystems, Darmstadt, Germany) with a Turbo spray ion source in negative ionization mode. The analyte parent ion → product ion for multiple reaction monitoring (MRM) were the following: *m/z* 263.0 →153.2 (collision energy (CE) -22 V; declustering potential (DP) -35 V) for abscisic acid; *m/z* 269.0 →159.2 (CE -22 V; DP -35 V) for D_6_-abscisic acid; *m/z* 209.1 →59.0 (CE -24 V; DP -35 V) for jasmonic acid; *m/z* 215.1 →59.0 (CE -24 V; DP -35 V) for D_6_-jasmonic acid; *m/z* 136.9 →93.0 (CE -22 V; DP -35 V) for salicylic acid; *m/z* 140.9 →97.0 (CE -22 V; DP -35 V) for D_4_-salicylic acid; *m/z* 290.9 → 165.1 (CE -24 V; DP -45 V) for *cis*-(+)-12-oxophytodienoic acid (*cis*-OPDA), *m/z* 322.2 →130.1 (CE -30 V; DP -50 V) for jasmonic acid-isoleucine conjugate; *m/z* 328.2 →136.1 (CE -30 V; DP -50 V) for jasmonic acid-^13^C_6_-isoleucine conjugate. For ABA quantification after drought stress the elution profile was modified as follows: 0–0.5 min, 10% B; 0.5–4.0 min, 10–90% B; 4.0–4.02 min 90–100% B; 4.02–4.5 min 100% B and 4.51–7.0 min 10% B keeping a flow rate of 1.1 mL/min.

### Genotyping

For genotyping, DNA of single leaves of 3-week-old plants was isolated according to a modified protocol of Konieczny and Ausubel [29]. Samples were ground as described above. Extraction was performed using half of the given volumes of chemicals and buffer. All centrifugation steps were carried out at 16000 x g. After precipitation, sample was centrifuged for 20 min and pellet was directly washed in 70% ethanol. The dried pellet was dissolved in 30 μL water to achieve a higher concentration. Genotyping primers were designed with SALK T-DNA primer design tool (http://signal.salk.edu/tdnaprimers.2.html). Sequences of the primers are listed in S1 Table. Native Taq DNA polymerase and 10 mM dNTP Mix (both Invitrogen^TM^, Darmstadt, Germany) were used for PCR. Mastermix was prepared according to the manufacturers’ protocol and scaled down to a reaction volume of 10 μL, including 1.5 μL of template.

### Semiquantitative reverse transcription (RT)-PCR

RNA isolation, DNase treatment and cDNA synthesis were carried out as described above. *ACTIN2* was used as housekeeping gene. The same primer sequences for *ACTIN2* were used as described before [17]. *CML9* primers were designed with the same tools as qRT-PCR primers (see S1 Table). PCR was performed as described above.

### Statistical analysis

Statistical significances were tested using t-test or Wilcoxon-test in RStudio 0.98.1103.0, or by ANOVA using SigmaPlot 12.2.0 (Systat Software GmbH, Erkrath, Germany). False discovery rate (FDR, [30]) was calculated if t-test or Wilcoxon-test was repeated more than 3 times in 1 data set. Statistical tests used for different experiments are indicated in the figure legends.

## Results

### Expression of *CML9* is induced upon herbivory

**Fig 1:**
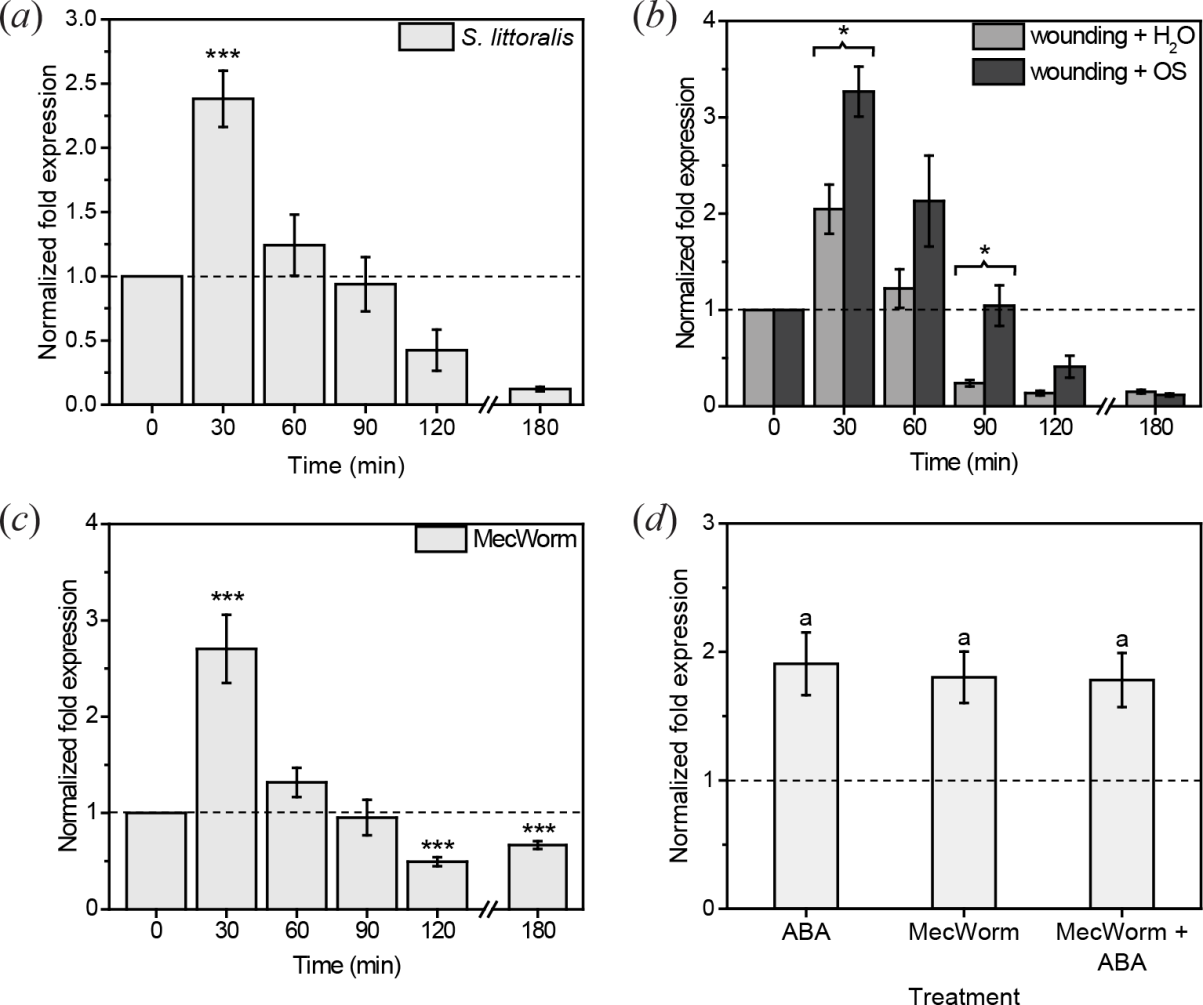
Expression of *CML9* in response to different herbivore-associated stimuli. Changes in *CML9* transcript level in *A*. *thaliana* wild type leaves (Col-0) upon *S*. *littoralis* feeding (*a*), mechanical wounding with a pattern wheel and application of either water or *S*. *littoralis* OS (*b*), mechanical damage by MecWorm (*c*) or ABA spray combined with MecWorm treatment (*d*) are plotted. Expression level in (*a*), (*b*) and (*c*) was determined after 30, 60, 90, 120 and 180 min of treatment. Untreated plants were used as controls. Fold expression in (*d*) was measured after 30 min of MecWorm treatment with a 60 min pre-incubation with 100 μM ABA solution or a 0.02% ethanol solution, or just incubation with ABA. Control plants were treated with 0.02% ethanol. The *CML9* fold expression was normalized with respect to the *RPS18B* transcript level and calculated relative to respective controls. Bars represent the means ± standard error (SE) (n ≥ 6 (*a*), n ≥ 5 (*b*), n ≥ 11 (*c*), n ≥ 10 (*d*)). Experiments were repeated at least two times independently. Statistically significant changes in expression levels were determined by one-sample Wilcoxon test (*a*) or one-sample t-test (*c*). Statistically significant differences between the different treatments were determined by unpaired two-sample Wilcoxon test at each time point separately (*b*) or by one way ANOVA (*d*). Asterisks indicate significances (* P < 0.05, *** P < 0.001). P-values for (*a*), (*b*) and (*c*) are FDR corrected. Letters in (d) indicate no statistic differences.

Several transcript studies in *Arabidopsis* already indicated that CML9 is induced by herbivore-associated stimuli [14, 17, 21]. Nevertheless, other data showed no change of the expression level after wounding or herbivory and even repression of the gene after application of methyl-JA [31]. In order to test these contradictory results, we treated the plants with various herbivore-associated stimuli and analyzed the *CML9* expression level using qRT-PCR. We found that *CML9* is significantly induced about 1.5-fold already after 30 min feeding of the chewing insect *S*. *littoralis* (Fig 1(*A*)). This induction was transient and reached the base level after 60 min, further reduction of the expression levels at 2 and 3 h was not significant. Since feeding of herbivores can be recognized by the mechanical wounding pattern as well as by elicitors in the OS of herbivores [32], we tested further if both stimuli can induce a change in *CML9* transcript level. Therefore, plants were mechanically wounded with a pattern wheel and either water or *S*. *littoralis*-derived OS was applied to the wounds. In both cases *CML9* was quickly and transiently upregulated (Fig 1(*B*)), as was found in the real insect treatment. After 30 min, wounding induced *CML9* one-fold, compared to the untreated controls. The application of insect OS increased *CML9* transcript level even two-fold compared to controls, suggesting that both stimuli cause the induction of *CML9* upon feeding of *S*. *littoralis* to a similar extend. Additionally, wounding treatment was repeated using MecWorm, a robotic larva mimicking the wounding pattern of a chewing insect [26], to confirm the results of the artificial wounding with a pattern wheel. MecWorm treatment caused the same expression pattern as all the treatments before (Fig 1(*C*)): *CML9* was induced about 1.5-fold shortly after wounding by MecWorm. This regulation was also transient and *CML9* transcript level decreased in later time points.

*CML9* was shown to be induced by ABA as well [21]. Regarding to the given crosstalk between the ABA pathway and the defense against herbivores [6], we tested if there is an additive or synergistic effect of both treatments together on the *CML9* expression level. We compared the expression level of plants treated only with MecWorm or ABA with plants that were treated with both (Fig 1(*D*)). All three treatments induced *CML9* expression to the same extends. These data exclude an additive effect of ABA and wounding by an herbivore on the induction of *CML9*.

### Herbivore performance is not affected by *CML9* knock-out or overexpression

**Fig 2:**
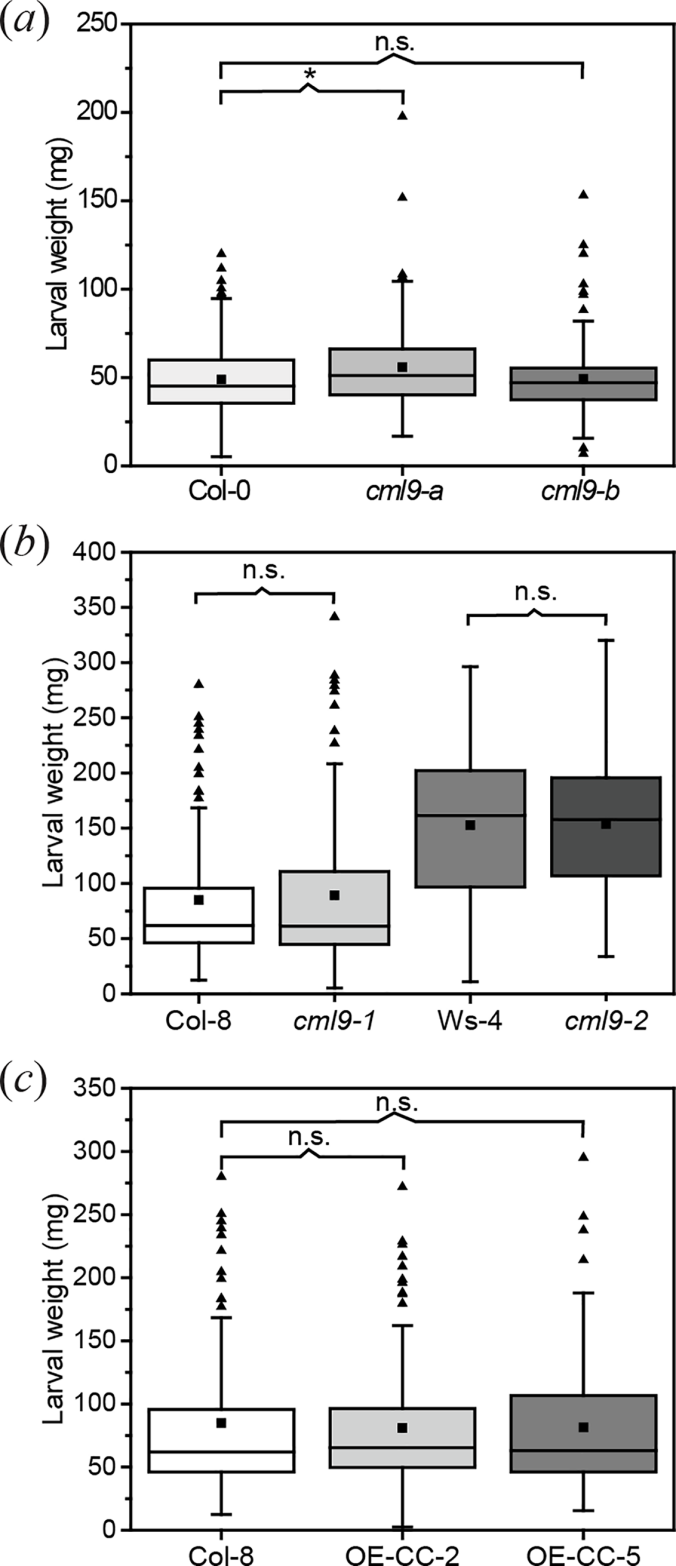
Susceptibility of different *Arabidopsis* mutant lines of *CML9* on *S*. *littoralis* feeding. Gain of larval weight was determined after feeding on Col-0 wild type plants and *cml9-a* and *cml9-b* knock-out lines (*a*), Col-8 and Ws-4 wild type plants and *cml9-1* and *cml9-2* knock-out lines (*b*) and Col-8 wild type plants and OE-CC-2 and OE-CC-5 overexpression lines (*c*) for one week. First instar larvae of *S*. *littoralis* were pre-weighed to reduce experimental variation. Three larvae were placed on each plant. After feeding period larval weight was determined. The boxplots show the distribution of the measured data. The box indicates the middle 50% of the data points. Black triangles represent outliers and the black squares the mean values. Whiskers are defined as 1.5 fold interquartile range (IQR). Experiments were repeated at least five times independently (n = 134 (Col-0), n = 133 (*cml9-a*), n = 139 (*cml9-b*), n = 98 (Col-8), n = 92 (*cml9-1*), n = 109 (Ws-4), n = 111 (*cml9-2*), n = 89 (OE-CC-2), n = 91 (OE-CC-5)). Statistically significant differences between larval weights of different genotypes were determined by unpaired two-sample Wilcoxon test. Asterisk indicates significance (* P < 0.05), n.s. means not significant.

Because of the fast induction of *CML9* after *S*. *littoralis* feeding, the functional relevance of CML9 to the plant defense against this herbivore was further investigated by studying *CML9* loss of function mutants. Two homozygous intronic T-DNA insertion lines (*cml9-a* and *cml9-b*) were used in a conventional one-week feeding assay. Feeding performance of the larvae was determined by measuring the larval weight (Fig 2). Larvae feeding on *cml9-a* gained little but significantly more weight than larvae feeding on corresponding wild type plants, although the measured effect was small. However, this result was not confirmed with the second knock-out line. Larvae feeding on *cml9-b* gained as much weight as on wild type plants (Col-0) (Fig 2(*A*)). Since the different response of these two loss-of-function mutants, two more knock-out mutant lines (*cml9-1* and *cml9-2* [21]) and two overexpression lines (OE-CC-2 and OE-CC-5[23]) were tested in feeding assays. All of the additional mutant lines display a different ecotype background: whereas *cml9-1* and both overexpression lines are in Col8 background, *cml9-2* is in Ws background. In general, *S*. *littoralis* larvae gained more weight on genotypes with the Ws ecotype than on those with the Col background (Fig 2(*B*)). However, the larvae gained as much weight on the knock-out lines as on the corresponding wild types, confirming the results of the *cml9-b* lines (Fig 2(*B*)). Moreover overexpression of CML9 did not influence the larval performance as well (Fig 2(*C*)).

**Fig 3:**
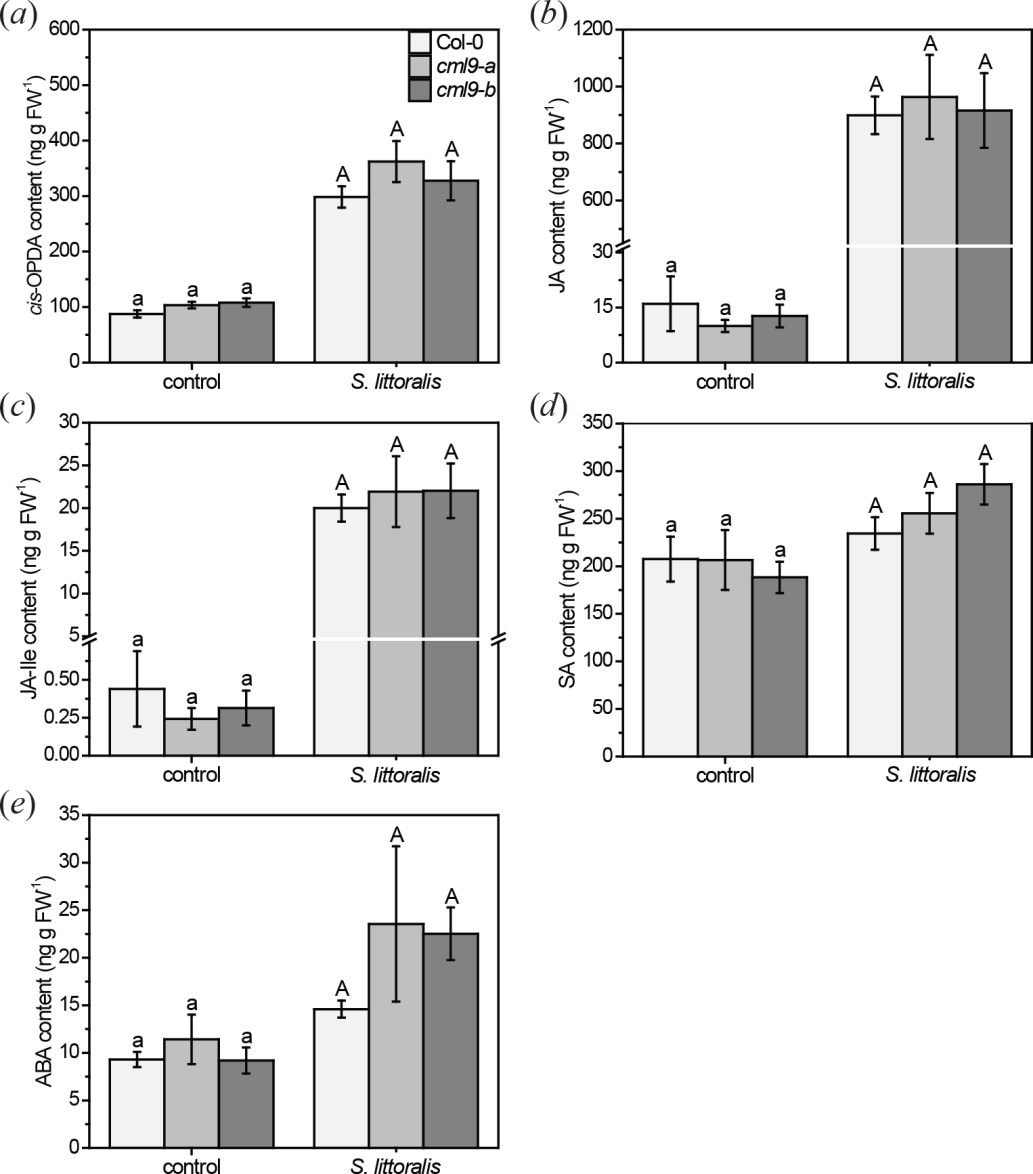
Phytohormone contents of *A*. *thaliana* wild type and *cml9* mutant plants after *S*. *littoralis* feeding. Levels of *cis*-OPDA (*a*), JA (*b*), JA-Ile (*c*), SA (*d*), ABA (*e*) after larval feeding for one week in ng g^-1^ fresh weight (FW). Phytohormones were extracted only from local fed leaves. Untreated plants were used as controls. Bars represent means ± SE. Experiment was repeated three times independently (n ≥ 13). Statistically significant differences between phytohormone content of different genotypes among one treatment were determined by Kruskal-Wallis one-way ANOVA on ranks, using Dunn’s method as post-hoc test. No significant differences were measured, as indicated by the letters. Legend for color code see (*a*).

To further investigate the different susceptibility of *cml9-a* and *cml9-b* to *S*. *littoralis* feeding, we measured the phytohormone content after feeding of the larvae. Especially the jasmonates are main regulators of this defense response [4]. Thus, we measured the content of JA, its precursor *cis*-12-oxophytodienoic acid (*cis*-OPDA) and the active jasmonate, jasmonic acid-isoleucine (JA-Ile) (Fig 3(*A*)–3(*C*)). All three jasmonates increased after feeding of *S*. *littoralis*, but to the same extend in both *cml9* lines as in Col-0 wild type plants. Besides, SA and ABA have been shown to modulate herbivore defense [6, 8, 9]. Hence, we analyzed the levels of these phytohormones additionally (Fig 3(*D*) and 3(*E*)). The ABA content was slightly increased upon insect feeding, whereas the SA level was nearly the same as in control plants. Nevertheless, there were no significant differences in the SA or ABA level obtained between the *cml9* lines and in comparison to the wild type. Both *cml9* lines show similar results for all tested phytohormones, thus slight differences observed in the feeding behavior of *S*. *littoralis* between the lines are not due to a change in phytohormone elevation.

**Fig 4:**
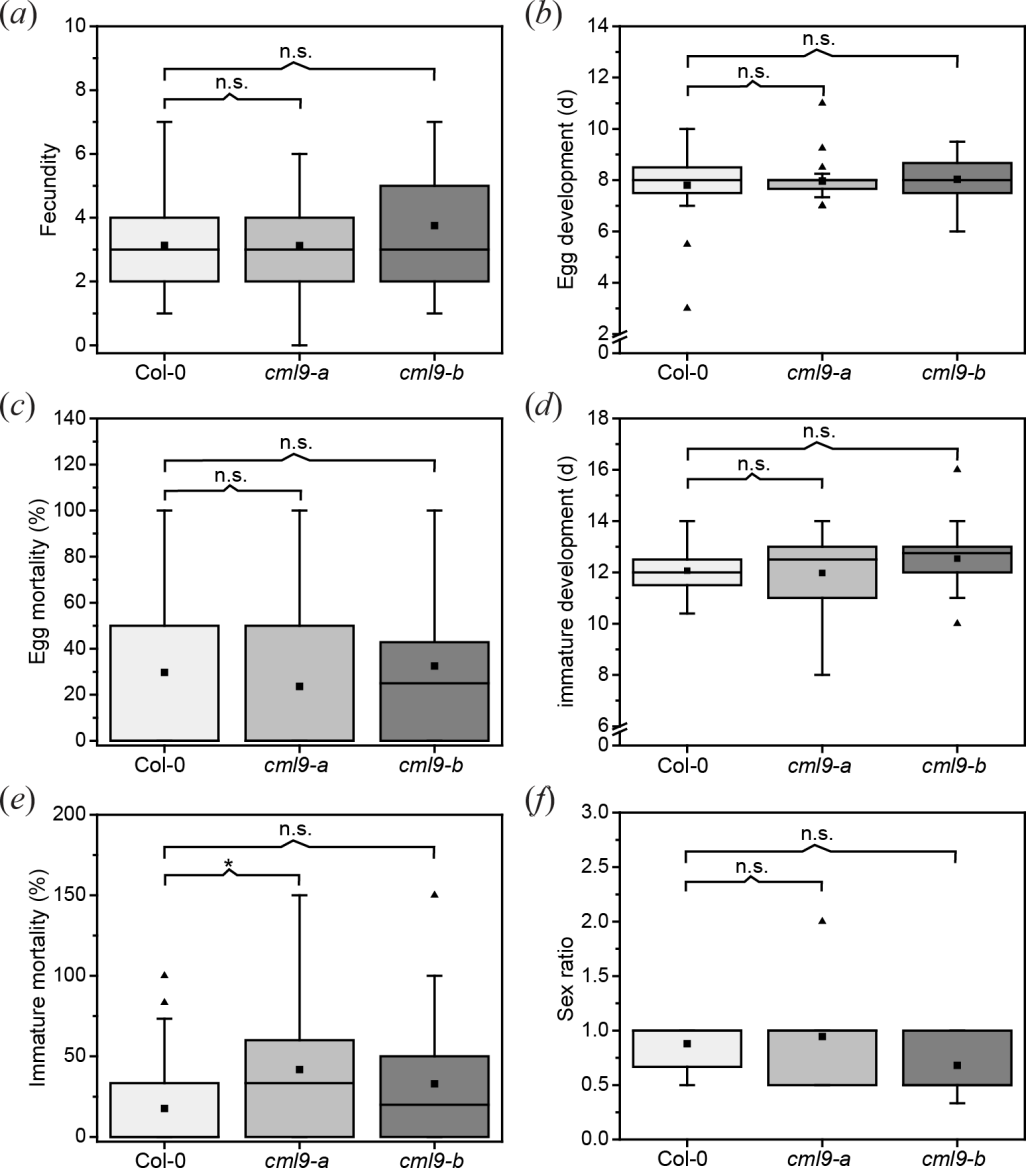
*T*. *urticae* performance on *cml9-a* and *cml9-b*. Different fitness parameter monitored over development of one generation of spider mites are shown in box plots: fecundity (number of eggs per female per 24 h) (*a*), egg development (means of all eggs on one plant) (*b*), egg morality (*c*), immature development (means of all immatures pooled together on one plant: larva, nymphochrysalis, protonymph, deutochrysalis, deutonymph, teleiochrysalis) (*d*), immature mortality (sum of mortality of all immature stages) (*e*), sex ratio (males : females) (*f*). The box indicates the middle 50% of the data points. At least n = 31 plants per genotype were used. Black triangles represent outliers and the black squares the means. Whiskers are defined as 1.5 fold IQR. Statistically significant differences between Col-0 wild type and mutant lines were determined by unpaired two-sample Wilcoxon test. Asterisk indicates significance (* P < 0.05), n.s. means not significant.

Regarding the data of the *S*. *littoralis* feeding performance on the *cml9* lines, we tested the performance of a second herbivore with a different feeding strategy on *cml9-a* and *cml9-b* mutants in comparison to wild type plants. We used the piercing-sucking spider mite *T*. *urticae*. Different fitness parameters of *T*. *urticae* were monitored daily over development of one generation (Fig 4). Spider mites established successfully on all plants and dispersed over the leaves. Infested plants showed numerous chlorotic spots. All tested fitness parameters in the early stage of spider mite development that we obtained on the *cml9-a* and *cml9-b* were comparable to those on Col-0 wild type plants, such as fecundity, time of egg development and the egg mortality. Even the time of immature development and the sex ratio were not changed in *cml9-a* and *cml9-b* plants compared to the Col-0 wild type. Only the immature mortality of *T*. *urticae* reared on *cml9-a* plants was little higher than on wild type plants. Collectively, these results suggest that CML9 is not a regulator of plant defense against the tested herbivores.

### *Arabidopsis thaliana cml9* plants show a wild type-like response to *Alternaria brassicicola*

**Fig 5:**
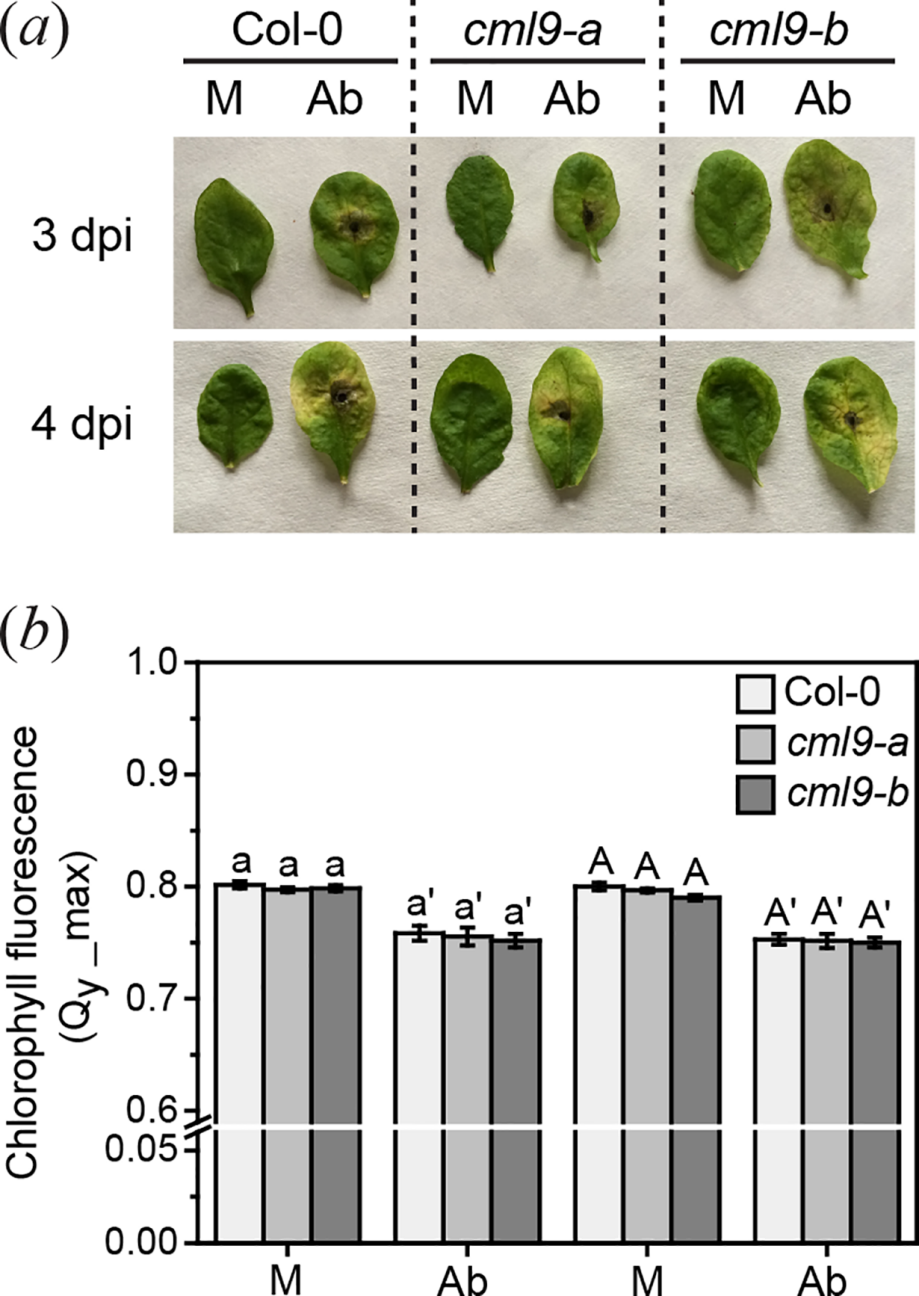
Response of *A*. *thaliana* wild type and *cml9* lines to *A*. *brassicicola* infection. Macroscopic observation of lesion formation (*a*) and measurement of chlorophyll fluorescence (*b*) of wild type and mutant leaves 3 and 4 day post-inoculation (dpi) with *A*. *brassicicola* spore suspension (Ab) or 0.01% Tween-20 solution as mock (M). Experiment was repeated three times independently. Plants shown are representative. Statistically significant differences in chlorophyll fluorescence of different genotypes among one treatment were determined by one-way ANOVA. No significant differences were measured, as indicated by the letters.

CML9 is described to modulate plant defense against different strains of the phytopathogenic bacteria *P*. *syringae* [23]. To further investigate if CML9 might act as a regulator in defense against other pathogens, we examined the reaction of *cml9-a* and *cml9-b* plants upon fungal infection with *A*. *brassicicola*. *A*. *thaliana* wild type Col-0 and *cml9-a* and *cml9-b* leaves were inoculated with a spore solution or mock treated and analyzed 3 and 4 days post-inoculation. Both *cml9* lines showed the same level of susceptibility than the wild type (Col-0) to the pathogen at tested time points (Fig 5(A)). There were no obvious differences in the formation and size of lesions at the macroscopic level observed. Additionally, we evaluated plant susceptibility by measuring chlorophyll fluorescence parameters of the inoculated leaves. In all plants the determined QY-max coefficient (0.75) indicated a decrease in fluorescence upon treatment with *A*. *brassicicola* (Fig 5(B)). Nevertheless, chlorophyll fluorescence in the treated mutant lines was reduced to the same extent like in infected wild type (Col-0) plants, confirming the phenotypic results. Hence, CML9 does not seem to modulate plant defense against *A*. *brassicicola*.

### Loss-of-function mutants are as susceptible to drought as wild type plants

**Fig 6:**
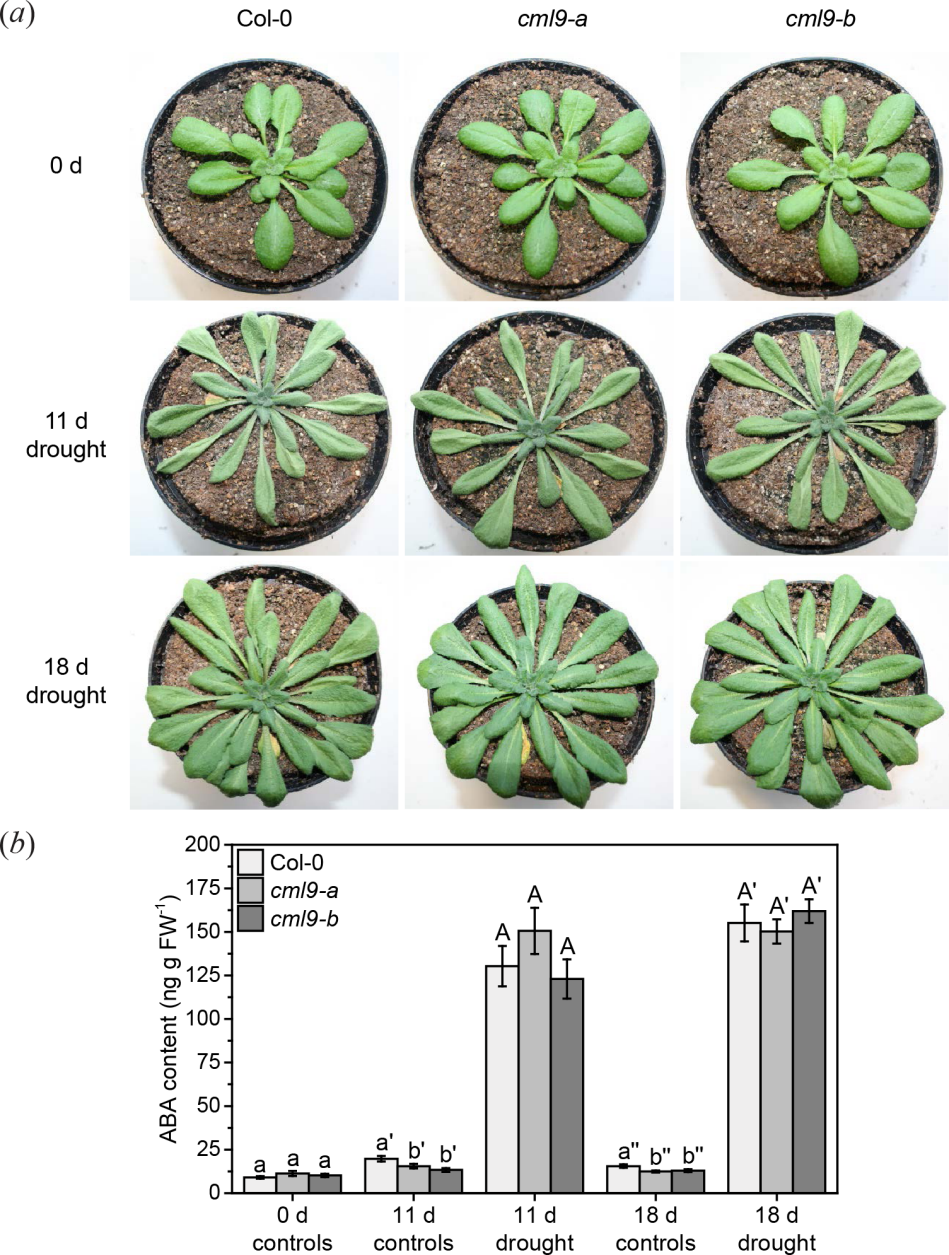
Comparison of drought stress response of wild type and *cml9* mutants. Representative pictures (*a*) and mean ABA level ± SE (*b*) of wild type, *cml9-a* and *cml9-b* before (0 d) and after drought (11 d and 18 d). Experiment was started with 4-week-old plants (0 d). Plants exposed to drought for 18 d were watered once after 11 d. All plants shown in (*a*) are independent from each other. Treatment was repeated 4 times independently (n = 20). Statistically significant differences between ABA content of different genotypes among one treatment were determined by one-way ANOVA, using Student-Newman-Keuls (SNK) method as post-hoc test. Different letters indicate significant changes (P < 0.05).

In previous studies it was shown that CML9 is very likely a negative regulator of ABA-related stress responses, e.g. drought [21]. It was hypothesized that the higher drought tolerance of *cml9* could be explained by hypersensitivity of the mutants to ABA, since both of the tested mutant lines showed a wild type-like ABA content upon drought [21]. Nevertheless, in that study ABA elevation was just analyzed after some hours of drought stress, but not in long term stress treatment like it was done for other CMLs by Scholz, Reichelt [19]. To elucidate whether a later increase in the ABA level could explain the higher resistance of *cml9* against drought, we kept the plants under drought conditions for 11 d or 18 d with watering once after 11 d. In contrast to previous published results, *cml9-a* and *cml9-b* mutants were as tolerant to drought as wild type plants (Fig 6(*A*)). Parallel to this stress treatment we quantified the ABA level in all genotypes. Without stress, all plants had similar endogenous ABA content (Fig 6(*B*)). As expected, a significant increase in this ABA content was observed in wild type (Col-0) 11 and 18 days after the beginning of the stress treatment (Fig 6(*B*)). Although we measured a slightly lower content of ABA at 11 d and 18 d in *cml9-a* and *cml9-b* control plants, the ABA elevation after drought showed the same profile and levels as the corresponding wild type line. Taken together, our data suggest that CML9 does not act as a key regulator of drought stress responses in plants.

### *cml9-a* and *cml9-b* differ genetically

**Fig 7:**
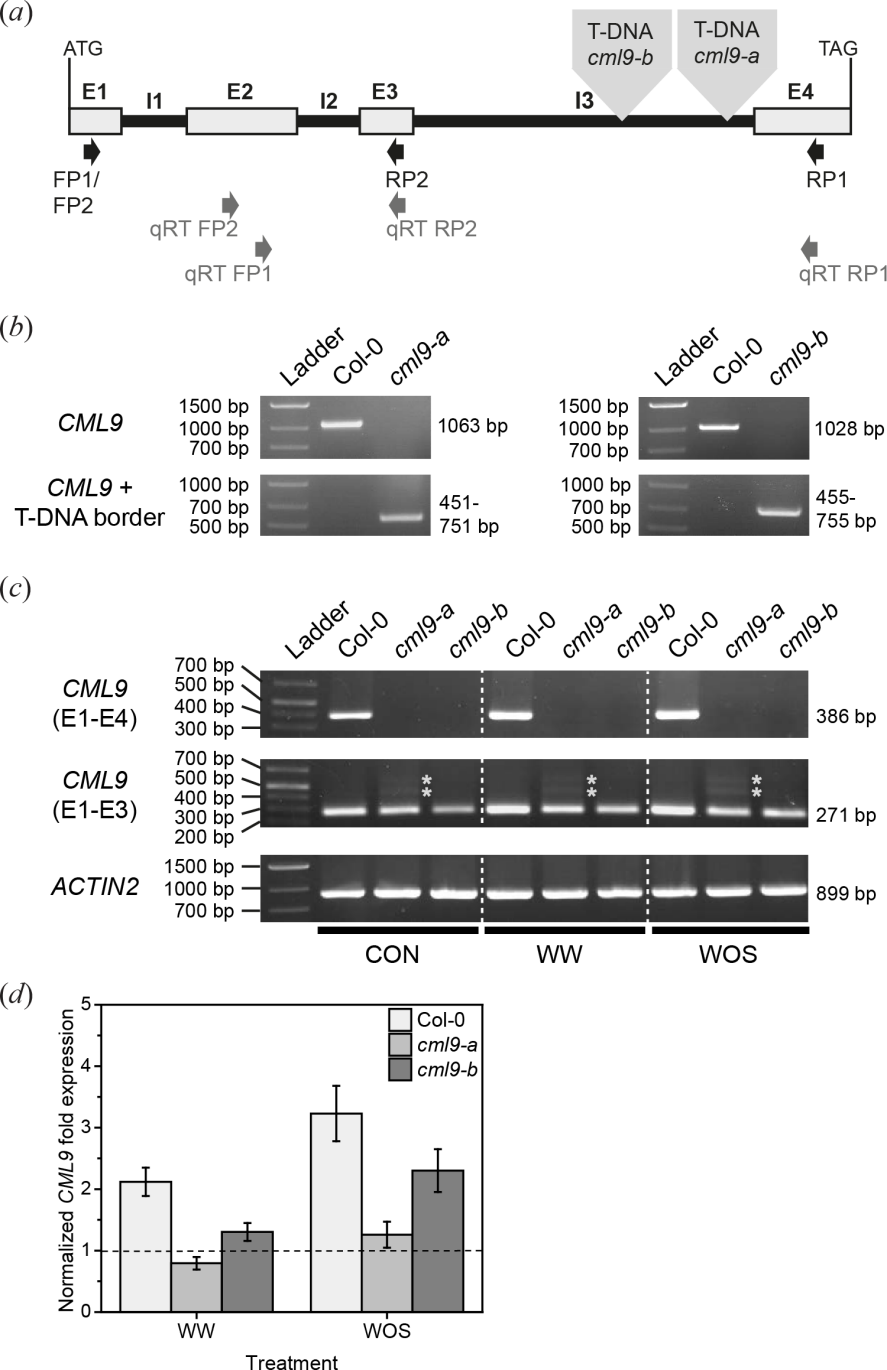
Genetic differences between *cml9-a* and *cml9-b*. (*a*) Schematic overview of *CML9* with T-DNA insertions and the used primers for RT- and qRT-PCR. Exons are indicated with E, introns with I. Light gray triangles indicate T-DNA insertions. RT primers used are indicated by black arrows, qRT primers by grey arrows (qRT FP1 and qRT RP1 are published as CML9 primers in Vadassery, Scholz [16]). Total length of *CML9* gDNA without insertions is 1137 bp. (*b*) Verification of T-DNA insertions in *CML9* by genotyping. The expected product length is indicated on the right sites of the respective pictures. (*c*) Semi quantitative RT-PCR analysis of *CML9* expression in wild type and knock-out mutants. Plants were treated with a pattern wheel and either water (WW) or *S*. *littoralis* oral secretion (WOS) was applied for 30 min. Untreated plants were used as controls (CON). Besides full length expression, expression of the E1-E3 fragment, upstream of the intronic T-DNA insertions, is shown. Expression of *ACTIN* was used as quantitative control. The expected product length is written on the right sites of the respective pictures. Asterisks indicate unspecific bands in the *cml9-a* mutant. (*d*) Normalized fold expression of *CML9* E1-E3 fragment in wild type and *cml9-a* and *cml9-b* lines. Plants were treated as described in (*c*). Expression level was normalized with respect to the *RPS18B* transcript level. Bars represent the means ± SE (n ≥ 5). Experiments were repeated two times independently. Statistically significant changes were determined by two-way ANOVA. Results of statistical analysis are shown in Table 1.

In this study two independent intronic T-DNA insertion lines were used as knock-out mutants, both with a T-DNA insertion in the third intron (Fig 7(*A*)). Prior to all experiments, the insertion of the T-DNA was confirmed by genotyping (Fig 7(*B*)). Two different primer pairs were used for PCR: one gene specific primer pair and a second one including the left border of the insertion. In both mutants there was no product for the intact *CML9* detectable (upper row, Fig 7(*B*)). Only a product consisting of truncated *CML9* and the T-DNA border was observed (lower row, Fig 7(*B*)). Both lines were verified as homozygous. The absence of the full length *CML9* transcript in MecWorm treated mutants was confirmed by semi quantitative RT-PCR (S1 Fig). Since these two *cml9* lines exhibited slightly different responses to herbivore treatments as described above, a more detailed genetic analysis for both lines was performed.

Although T-DNA insertion mutants are usually very stable, the use of intronic insertion lines can be problematic. It was shown that different environmental stimuli can cause alternative splicing of introns leading to a loss of the T-DNA insertion and, thus, to a wild type-like expression of the gene [33, 34]. In order to examine whether herbivory by *S*. *littoralis* is able to stimulate such an alternative splicing event in the two *cml9* lines, we analyzed the *cml9* gene expression by another semi quantitative RT-PCR. Since the first RT-PCR (S1 Fig) revealed that mechanical wounding by the larvae is not leading to any *CML9* transcript accumulation in the mutants, we investigated if treatment with the insect-derived OS may stimulate gene expression. The OS treatment was performed as described above. No *CML9* transcript was detectable in both mutants upon application of OS ((Fig 7(*C*)), upper row). Furthermore, the same was observed for the water treatment, confirming the result of the first RT-PCR. Thus, a loss of the entire T-DNA insertion by alternative splicing due to herbivory is unlikely for both *cml9* mutant lines.

The expression of fragments upstream or downstream of a T-DNA insertion can lead to the production of a truncated protein [35]. Just recently it has been shown, that two T-DNA alleles of a receptor kinase respond differently, due to the production of a truncated protein in one of these lines [36]. To exclude this possibility, the expression of the fragment upstream of the T-DNA insertion (from the first to the third exon) was investigated by RT-PCR. In all treatments and controls in the mutants, this fragment was expressed ((Fig 7(*C*)), middle row). In *cml9-a* plants, two additional unspecific bands were detectable. Sequencing of these fragments revealed that they are artefacts of an incorrect splicing event. The sequences or part of the sequences of the first and second intron are still included in the products. Thus, the T-DNA insertion in *cml9-a* seems to influence the splicing process of the *CML9* RNA, suggesting that it is unlikely that a truncated, but functional protein is produced. In *cml9-b* only the correctly spliced fragment was found. If a truncated protein was produced, two of the four EF-hands would miss, leading to a lower Ca^2+^ binding capacity and probably to reduced activities.

When comparing the expression of the expected *CML9* fragment with the house keeping gene expression ((Fig 7(*C*)), lower row), the transcript level of the fragment was lower in both mutants than in the wildtype. To refine this observation a qRT-PCR was performed (Fig 7(*D*)). Data were analyzed by two-way ANOVA, in order to test whether there is a difference among the treatments and the genotypes and if one of those could be explained by the other. The statistical analysis revealed that there is a significant increase in the fragment transcript abundance upon OS treatment in all three genotypes (Table 1). Nevertheless, even when a very low threshold of two-fold was used, upon both treatments an induction of the fragment could only be observed in the wild type. In *cml9-b* the fragment is only induced upon OS treatment, whereas in *cml9-a* the fragment is not induced at all. Furthermore there is a significant difference between the genotypes. However, this statistical difference between the genotypes was not due to the treatments. This suggests that the two *cml9* lines vary genetically and this also might explain some varying results in herbivore treatments.

**Table 1:** Statistical analysis of qRT PCR of exon1-exon3 fragment of *CML9*. Data were log-transformed and analyzed by two-way ANOVA. SNK was used as post hoc test (P < 0.05). DF = Degrees of Freedom.

Tested Variables	Results two way ANOVA			Results SNK
	P	F	DF	
Treatment	<0.001	17.681	1	WW < WOS
Genotype	<0.001	26.179	2	*cml9-a* < *cml9-b* < wild type
Treatment x Genotype	0.846	0.168	2	-

## Discussion

In plants, the perception of environmental stimuli is followed by a fast calcium elevation inside the cells [10]. These calcium signals encode information about the stimulus that need to be translated into the appropriate response [11]. However, relatively little is known about the decoding of such calcium signals. CML proteins are important in sensing calcium signals after various external stimuli. Here we focused on the CML9. By using different CML9 mutant lines, we investigated the role of this calcium sensor in biotic and abiotic stress responses.

### CML9 is not a regulator of plant herbivore defense

CML9 was described as a regulator of the plant defense against phytopathogenic bacteria [23]. The signaling cascade after recognition of a pathogen is also related to the signaling pathway after herbivory [32]. Thus, we examined if CML9 is also involved in the herbivore defense. First, we demonstrated that *CML9* is induced upon feeding by the insect herbivore *S*. *littoralis* (Fig 1(*A*)). This enhancement in the transcript level is caused by the mechanical wounding of the larvae as well as by the OS (Fig 1(*B*) and 1(*C*)). Unlike the known defense regulators *CML37* and *CML42* that are mainly regulated by one of the two stimuli [17, 18], *CML9* is equally induced by both. The result that *CML9* is a wound-inducible gene is of great interest, because the yet published literature was contradictory [21, 31]. Besides, our data indicate that the induction of the *CML9* transcript level after wounding is not synergistically regulated by ABA. Although *CML9* expression was upregulated by either of the treatments, ABA had no additional effect on the transcript level (Fig 1(*D*)). This suggests that the regulation of *CML9* mRNA levels after herbivory might be independent of ABA. Among all herbivore-associated treatments *CML9* displayed a characteristic expression dynamic. Like *CML42*, *CML9* expression was fast and transiently up-regulated and down-regulated at later time points. This result was quite surprising, since it was described that *CML9* expression occured late and remained high after OS treatment [16]. On the other hand, the fast and transient expression profile seems to be typical for *CML9*. The same dynamics were found after drought and pathogen-associated stress treatments [21, 23]. Compared to the stimulation of *CML37* and *CML42* mRNA levels after herbivory, the *CML9* transcript is only slightly induced [17, 18]. The same holds true for the *CML9* mRNA level after pathogen treatments [23]. Moreover, semi quantitative RT-PCR revealed that the basic level of the *CML9* transcript in untreated plants is already quite high (Fig 7(*C*)), suggesting that *CML9* is rather constitutively expressed than strongly induced.

Despite the fast upregulation of *CML9* after herbivore treatment, the analysis of different *CML9* knock-out lines revealed that CML9 is not a key player in herbivore defense. In three independent lines (*cml9-b*, *cml9-1*, *cml9-2*) the performance of the chewing insect *S*. *littoralis* was unaffected by the loss of function of the gene (Fig 2*A and 2B*). Similar results were obtained for the performance of the piercing-sucking spider mite *T*. *urticae* on the knock-out line *cml9-b* (Fig 4). Only in *cml9-a* line, slight changes in the herbivore performance were observed. In detail, *T*. *urticae* displayed a higher immature mortality (Fig 4), which represents one out of six examined parameters. On the other hand, *S*. *littoralis* larvae performed better on the *cml9-a* line than on the Col-0 wild type (Fig 2(*A*)), suggesting a positive influence of CML9. However, compared to loss-of-function mutants of the positive defense regulator CML37 [18], *cml9-a* line was only slightly more susceptible to *S*. *littoralis* and more similar to the three additional knock-out lines tested (Fig 2(*A*) *and* 2(*B*)). As treatment with *T*. *urticae* showed similar results (Fig 4), all these data strongly suggest that the effects in *cml9-a* are not due to the loss-of-function of *CML9*.

Besides, throughout all feeding assays with *S*. *littoralis*, larvae gained more weight on plants with the Ws-4 ecotype background than on plants with Col-8 background (Fig 2(*B*)). This result is in agreement with previous studies showing that different insect species prefer Ws-0 to Col-0 ecotypes [37, 38] and could be correlated with lower glucosinolate content in the Ws-0 ecotype [37], a finding that also explains why the larvae fed better on Ws-4 lines (Fig 2(*B*)). Nevertheless, when using plants with different ecotype background for insect assays this difference between Col and Ws ecotypes should be taken into account.

Further analysis of *cml9-a* and *cml9-b* showed that both lines differ genetically (Fig 7(*C*) and 7(*D*)) that might explain the little variances in the herbivore treatment observed. Moreover, knock-out of *CML9* does not lead to a change in the phytohormone response to *S*. *littoralis* feeding (Fig 3) indicating once more that CML9 is not a key player in herbivore defense regulation.

In *Arabidopsis*, 50 CMLs are known and among them eight are regulated upon *S*. *littoralis* herbivory [16–18, 31]. Thus, it is conceivable that some of these proteins have redundant or overlapping functions. Inactivation of one of them does not necessarily have a great impact on a particular plant stress response. For example, the *cml24* but not *cml23* mutant shows under certain conditions a phenotype that differs from the wild type; however, in the *cml24*x*cml23* double mutant the *cml24* effect is modulated [39]. To exclude this scenario in case of CML9, *CML9* overexpression lines were examined. Both tested lines were as susceptible to *S*. *littoralis* feeding as their corresponding wild type, supporting the conclusion, that CML9 does not play any role in the defense against this herbivore.

Taken the results of gene expression data and the mutant analysis together, we suggest further to interprete gene regulations more cautiously. Even though *CML9* is significantly upregulated after herbivore treatment, it is not relevant for the defense response.

### CML9 is not regulating defense against necrotrophs

In Arabidopsis 50% of the upregulated genes upon *A*. *brassicicola* infection are also induced upon *P*. *syringae* treatment, although the response to both microbes is mediated via different pathways [40]. Because CML9 is described as mediator of the defense against the biotrophic bacterial pathogen *P*. *syringae* [23], we also examined its role in the defense against the necrotrophic fungal pathogen *A*. *brassicicola*. We found that CML9 has no functional relevance in the plant immune response to *A*. *brassicicola*. Both mutant lines, *cml9-a* and *cml9-b*, were as susceptible to the fungus as to Col-0 wild type plants (Fig 5). The defense against this fungus is mainly regulated by jasmonates like the defense against herbivores [3, 41], while the response to *P*. *syringae* is mediated mainly by SA [3]. Thus, our results are in one line with the results obtained in the herbivore assays and suggest that CML9 is also not regulating the defense against this necrotrophic pathogen. Hence, the different impact of CML9 on both pathogens might be explained by their different lifestyles that trigger different signaling pathways. Another explanation could be that CML9 is only coordinating the response to bacterial but not to fungal pathogens. The fact that CML9 has been shown to contribute to plant defense against bacteria mainly through a flagellin dependent pathway would favor this hypothesis [23]. Additional experiments with other pathogens with different lifestyle or virulence strategies will help to better position CML9 in plant defense pathways.

### CML9 does not mediate drought stress tolerance in general

In contrast to previous studies, our data suggest that CML9 is not a common regulator of drought stress. It was reported earlier that CML9 negatively regulates the drought response [21], but under our experimental conditions both *cml9a* and *b* mutants displayed the same behavior as the wild type (Col-0) upon drought treatment (Fig 6(*A*)). Consistent with this observation, no significant difference in the kinetics and level of ABA elevation was observed between drought stress-exposed mutants and the wild type plants (Fig 6(*B*)). Thus, it is unlikely that CML9 plays a key role in the drought stress response. The divergent results of our and previous studies might be caused by different experimental setups: while our plants were grown in single pots during the drought stress, many plants were cultivated together in one pot in the previous study, a situation that can cause intra- and interspecific competition [21]. Furthermore we did our stress treatment under short day conditions while the other setup was done under long day conditions. It could well be that the role of CML9 in drought stress response is dependent on the length of the photoperiod. However, our data indicate that CML9 seems not to be a general regulator of the plant drought stress response.

## Conclusion

Here, we investigated in more detail the role of CML9 in plant stress responses. CML9 was described to act as a calcium sensor at the crossroads of different pathways, like pathogen defense and abiotic stress responses [21, 23]. Our study specifies the known functions of CML9. Based on our data, CML9 does not regulate the plant drought stress response in general as otherwise suggested [21], but might have a role under certain conditions. It also does not regulate plant defense against herbivores, neither against chewing lepidopteran larvae nor against piercing-sucking spider mites. We further showed that knock-out of *CML9* does not affect the response of the plant to the fungal pathogen *A*. *brassicicola*. These results suggest that CML9 is not a general regulator of plant pathogen defense, but very likely specialized on defense against pathogenic bacteria or (hemi)biotrophic pathogens like *P*. *syringae* [23]. Moreover, the results of both the herbivore and pathogen assays further suggest that CML9 does not regulate jasmonate-mediated pathways at all. Therefore, we propose that CML9 should not be included in a group of CMLs that have a general role in plant stress regulation.

## Supporting information

S1 Table Primers used for different PCRs. (DOCX)

S1 Fig Verification of *cml9-a* and *cml9-b*. Semi quantitative RT-PCR analysis of *CML9* full transcript expression in wild type and knock-out mutants after 30 min of MecWorm treatment. Expression of *ACTIN2* was used as quantitative control. Water was used as negative control. The expected product length is written on the right sites of the respective pictures.
(DOCX)
