# Supplementary material for: Herbivory-responsive calmodulin-like protein CML9 does not guide jasmonate-mediated defenses in Arabidopsis thaliana
Source: PLoS One. 2018 May 16;13(5):e0197633. doi: 10.1371/journal.pone.0197633 (PMC5955546; doi:10.1371/journal.pone.0197633)
Supplement: S1 Table — (DOCX) [file pone.0197633.s001.docx]

**S1 Table.** **Primers used for different PCRs.**

| Primer name | Sequence | PCR |
| --- | --- | --- |
| LBb1.3 | 5´-ATTTTGCCGATTTCGGAAC-3´ | PCR Genotyping |
| *cml9-a* LP | 5´-TGAGCGATGTTGACATCTTTG-3´ | PCR Genotyping |
| *cml9-a* RP | 5´-TTTGGTTTGGTTCGAATTTTG-3´ | PCR Genotyping |
| *cml9-b* LP | 5´-GAATCGATCGGTTTGATGATG-3´ | PCR Genotyping |
| *cml9-b* RP | 5´-CATGGCATTTCACAAAATGC-3´ | PCR Genotyping |
| FP1/FP2 | 5´-CAGATCCAAGAGTTTTACGAAGCC-3´ | RT-PCR |
| RP1 | 5´-AGAAAACCATCGCCATCAAGG-3´ | RT-PCR |
| RP2 | 5´-CATCTCCGTCTCTGTCGAAC-3´ | RT-PCR |
| RPS18B F^a^ | 5′- GTCTCCAATGCCCTTGACAT -3′ | qRT-PCR |
| RPS18B R^a^ | 5′- TCTTTCCTCTGCGACCAGTT -3′ | qRT-PCR |
| qRT FP1^b^ | 5′- TTGGCAACGGTGGCATCACT -3′ | qRT-PCR |
| qRT RP1^b^ | 5′- CCATCGCCATCAAGGTCGGCT -3′ | qRT-PCR |
| qRT FP2 | 5´-CCAAAGGCGGAACAACTGCA-3´ | qRT-PCR |
| qRT RP2 | 5´-ACCATCTCCGTCTCTGTCGAACAC-3´ | qRT-PCR |

**^a^** Ref [16]; **^b^** Ref [16, published as CML9]
