# Supplementary material for: Herbivory-responsive calmodulin-like protein CML9 does not guide jasmonate-mediated defenses in Arabidopsis thaliana
Source: PLoS One. 2018 May 16;13(5):e0197633. doi: 10.1371/journal.pone.0197633 (PMC5955546; doi:10.1371/journal.pone.0197633)
Supplement: S1 Fig — Semi quantitative RT-PCR analysis of CML9 full transcript expression in wild type and knock-out mutants after 30 min of MecWorm treatment. Expression of ACTIN2 was used as quantitative control. Water was used as negative control. The expected product length is written on the right sites of the respective pictures. (DOCX) [file pone.0197633.s002.docx]

Ladder

*cml9-a*

Water

*cml9-b*

wild type


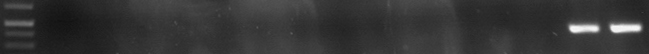

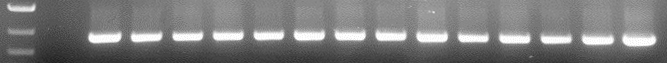


700 bp

500 bp

400 bp

300 bp

*CML9*

386 bp

1500 bp

1000 bp

700 bp

*ACTIN2*

899 bp

**S1 Fig.: Verification of *cml9-a* and *cml9-b.*** Semi quantitative RT-PCR analysis of *CML9* full transcript expression in wild type and knock-out mutants after 30 min of MecWorm treatment. Expression of *ACTIN2* was used as quantitative control. Water was used as negative control. The expected product length is written on the right sites of the respective pictures.
